# Protocol for single-cell spatial transcriptomic profiling of cultured cells and engineered tissues without embedding or sectioning

**DOI:** 10.1016/j.xpro.2025.104074

**Published:** 2025-09-11

**Authors:** Haylie R. Helms, Luiz E. Bertassoni

**Affiliations:** 1 Department of Biomedical Engineering, School of Medicine, Oregon Health and Science University, Portland, OR 97201, USA; 2 Knight Cancer Precision Biofabrication Hub, Knight Cancer Institute, Oregon Health and Science University, Portland, OR 97201, USA; 3 Cancer Early Detection Advanced Research Center, Knight Cancer Institute, Oregon Health and Science University, Portland, OR 97201, USA; 4 Division of Oncological Sciences, School of Medicine, Oregon Health and Science University, Portland, OR 97201, USA; 5 Division of Biomaterials and Integrative Biosciences, School of Dentistry, Oregon Health and Science University, Portland, OR 97201, USA

**Keywords:** Sequence analysis, Cell Biology, Cell culture, Single Cell, Cell-based Assays, Cancer, Genomics, Sequencing, Microscopy, Gene Expression, Tissue Engineering, Biotechnology and bioengineering

## Abstract

Spatial transcriptomics is a powerful tool for investigating how cellular composition and spatial relationships influence cell behavior. Here, we present a protocol for spatial transcriptomic profiling of 2D engineered tissues and cell cultures that are not compatible with standard embedding and sectioning. We detail steps for sample generation, fixation, and staining to integrate with Visium HD spatial technology. This protocol provides a flexible solution for sample generation for spatial transcriptomic analysis.

## Highlights

• Instructions for sample generation on a standard microscope slide coated with hydrogel
• Steps to fix and permeabilize sample in place for compatibility with Visium HD
• Generates a Visium HD library for whole-transcriptome, single-cell spatial analysis
• Guidance on processing FASTQ files with Space Ranger and initial data visualization

## Abstract (editor highlights)

Publisher’s note: Undertaking any experimental protocol requires adherence to local institutional guidelines for laboratory safety and ethics.

## Graphical abstract

**Figure:**
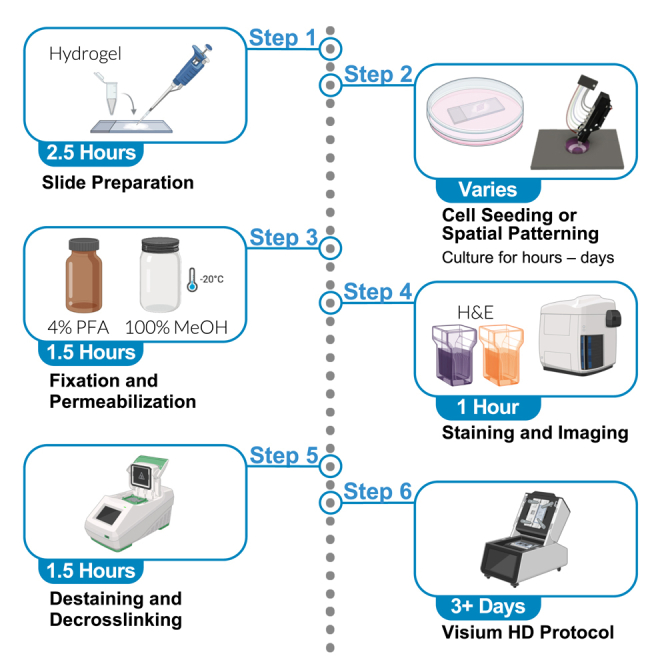

## Before you begin

Spatial transcriptomic (ST) analysis of tissues provides important insights into cell-cell interactions driving health and disease. However, the ability to interpret these interactions is often limited by patient-to-patient and sample-to-sample variability, as well as the observational nature of retrospective analyses, which makes it challenging to distinguish correlation from causation. Interpreting ST data is further complicated by difficulties in cell type annotation.^1^ Sparse transcript detection, lack of uniquely expressed genes, cellular heterogeneity, and cell state plasticity make assigning identities to each capture location difficult. While large cohorts and well-annotated reference atlases can help address these limitations, generating such resources is often hindered by high costs and limited sample availability.

In vitro cultures and engineered tissues offer a powerful alternative, enabling controlled experiments with defined cell populations, adjustable spatial organization, and multiple replicates for mechanistic insight. Furthermore, annotation challenges can be addressed by transducing cells with unique reporters prior to experimentation to facilitate identification. However, current commercially available ST technologies have been limited to standard formalin-fixed paraffin-embedded (FFPE), fixed frozen (FxF), or fresh frozen (FF) samples cut at a prescribed thickness, generally 5–10 μm. Sectioning has been a crucial step in spatial transcriptomics protocols to expose mRNA for probe hybridization. While it is possible to fix and embed 2D in vitro cultures, it is extremely difficult to obtain an even 5–10 μm thick section through a plane of cells that are only 3–10 μm thick. This creates a major barrier for profiling plated cells or engineered tissues generated using planar biofabrication techniques, such as tissue microfabrication^2^ and patterning.^3^ Cells that are cultured on conventional culture plates or seeded onto engineered spatially patterned hydrogels have been a staple of mechanistic cell biology.^4^^,^^5^^,^^6^^,^^7^ Moreover, as the field rapidly evolves toward single-cell resolution whole transcriptomic analyses, protocols that enable this level of characterization on planar cultured cells becomes increasingly necessary.

To overcome these problems, we present a method in which adherent cells are cultured on a standard microscope slide treated with collagen, fixed in place, and permeabilized for compatibility with Visium HD (10x Genomics). Visium HD is a whole transcriptome, sequencing-based platform in which probes hybridize to mRNA in a tissue section on a standard microscope slide. After probe ligation, the probes are transferred from the cells to the Visium HD capture slide using the CytAssist instrument (10x Genomics). The capture slide has a continuous lawn of 2 μm x 2 μm barcoded spots, enabling single-cell resolution during analysis. While the method is demonstrated here using a single-cell bioprinted 2D tissue,^8^ cells can be seeded onto the microscope slide following standard cell culture practices. We also envision that this protocol will be compatible with various biofabrication techniques, including, but not limited to, microfabrication for controlled patterning, microwells/chambers to prevent sample-to-sample communication, and live cell imaging with functional reporters.

### Innovation

This protocol modifies the 10x Genomics Visium HD Spatial Gene Expression workflow to enable compatibility with adherent cell cultures and engineered planar tissues. The commercial assay requires formalin-fixed paraffin-embedded, fixed frozen, or fresh frozen tissue sectioned at 5–10 μm, a step that is technically impractical for a single layer of cells. Our approach bypasses embedding and sectioning by growing cells directly on the required microscope slide, eliminating the need for sample transfer, followed by fixation and permeabilization, eliminating the need to section through the cells to expose the mRNA. The standard Visium HD protocol is then performed without modification, treating the cells or engineered tissue like a native tissue. This approach preserves the spatial arrangement of cells, unlike single-cell RNA sequencing, and avoids sample loss or damage during sectioning. It also expands the use of Visium HD beyond conventional tissue sections, allowing researchers to apply spatial transcriptomics to customizable and reproducible in vitro systems for precise mechanistic interrogation of spatially driven biological processes.

### Microscope slide sterilization

**Timing: 35 min**

**CRITICAL:** Without proper sterilization, the samples will become contaminated and the protocol will need to be aborted. Complete the following steps within a biosafety cabinet.

1. Sterilize a minimum of two microscope slides per Visium HD capture area by submerging in 70% ethanol for 30 min at room temperature in a sterile biosafety cabinet.

***Note:*** A Visium HD slide has two capture areas. Visium capture slides cannot be reused; therefore, if only one capture region is used the second capture region will be lost.

2. Remove the microscope slides from ethanol and allow liquid to evaporate. Thoroughly wash the slides to remove any residue by fully submerging in sterile distilled water; discard and replace the water with fresh sterile distilled water five times.
3. Transfer slides to a sterile 100 mm petri dish with a lid and proceed to step 1 of the protocol.

### Capture region reference guide

**Timing: 15 min**

**Figure 1:**
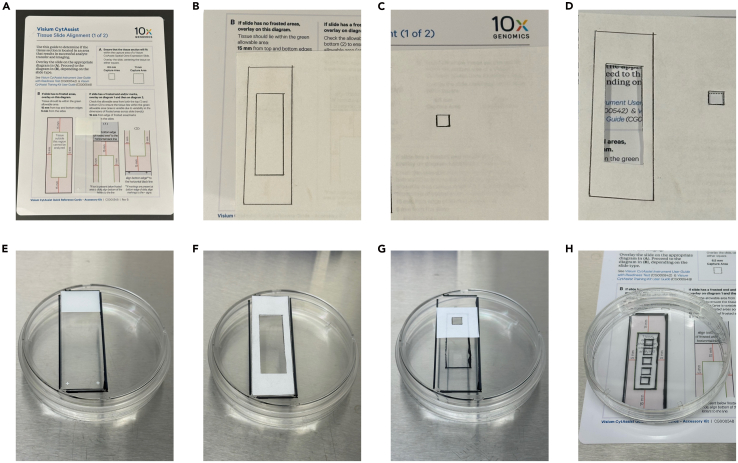
Creation of a reference guide on the outside of a culture dish to visualize viable probe transfer regions for the CytAssist (A) Visium CytAssist Tissue Slide Alignment Quick Reference Card, at scale with a microscope slide. (B) Viable transfer zone outline. (C) 6.5 x 6.5 mm capture area outline. (D) Stencils generated by cutting out regions of interest from B and C. (E) Alignment of the slide with the edge of the dish. (F) Viable transfer zone. (G) 6.5 mm capture area. (H) Final product, annotations on the bottom of the dish that fit the CytAssist slide alignment criteria.

This optional step creates a rough reference visual for where to treat the glass and place the sample so that there are no problems with sample access downstream during the CytAssist probe transfer and capture (Figure 1).

4. Obtain the Visium CytAssist Tissue Slide Alignment Quick Reference Card (CG000548) from the CytAssist which denotes the viable region of the microscope slide for probe transfer (Figure 1A).
5. Place a blank piece of paper on top of the Quick Reference Card and trace the viable region and slide outline shown in Section B with a fine tip pencil/pen (Figure 1B).
6. Move the paper to Section A and trace the 6.5 mm capture region box in an unused area of the paper (Figure 1C).
7. Use a razor blade (or equivalent) to cut along the viable region and capture region lines. (Figure 1D).
8. Spray a blank, nonsterile microscope slide with 70% ethanol and place in biosafety cabinet.
9. Place a sterile, capped, 100 mm petri dish upside down in the biosafety cabinet.
10. Place the microscope slide on the outside bottom surface of the petri dish and align it with the edge. Trace along the outside of the slide with a lab safe marker (Figure 1E).
11. Align the outer edges of the paper stencil with the slide outline. Trace the allowable capture region (Figure 1F).
12. Align the 6.5 mm capture region box stencil within the viable region and trace the boundary (Figure 1G).

***Note:*** Only one capture region can be transferred and analyzed per microscope slide. If cell numbers or labor is not of concern, we recommend preparing multiple capture regions (Figure 1H) and selecting the preferred sample to move forward with after viewing the endpoint H&E staining.

***Note:*** We do not recommend drawing fiducial marks directly onto the microscope slide which the sample will be generated on. Markings on the slide can interfere with the CytAssist alignment and transfer process. Additionally, the slide will be submerged in multiple solutions that can wash away fiducials.

### Cell culture

**Timing: variable**

13. Culture and expand selected cells following recommended protocols.

***Note:*** The amount of cells needed will depend on the experiment design. For patterning via single-cell bioprinting we recommend 1–2 × 10^6^ cells of each cell type for ease of handling.

## Key resources table

**Table:** 

REAGENT or RESOURCE	SOURCE	IDENTIFIER
**Chemicals, peptides, and recombinant proteins**		

1× Phosphate-buffered saline	Gibco	14190094
10× Phosphate-buffered saline	Thermo Fisher Scientific	AM9624
Bluing Reagent, Dako	Agilent	CS70230-2
Buffer EB	QIAGEN	19086
Cell culture medium (specific to cells of interest)		N/A
Eosin Y-solution, alcoholic	MilliporeSigma	HT110116
Ethanol, pure (200 Proof, anhydrous)	MilliporeSigma	E7023-500ML
Gill II Hematoxylin	Sigma	GHS232
Glycerol	Acros Organics	327255000
HCl, 0.1 N	Fisher Chemical	SA54-1
KAPA SYBR FAST qPCR Master Mix (2×)	Thermo Fisher Scientific	50-196-5281
Methanol, for HPLC	Millipore Sigma	34860
Milli-Q water		N/A
Nuclease-free water (not DEPC-treated)	Thermo Fisher Scientific	AM9937
Paraformaldehyde, 16% aqueous solution, EM grade	Electron Microscopy Sciences	15710
Potassium hydroxide solution, 8 M	MilliporeSigma	P4494-50ML
Primocin	InvivoGen	ant-pm-05
PureCol, type I bovine collagen solution	Advanced Biomatrix	5005-100ML
SSC Buffer 20× concentrate	MilliporeSigma	S66391L
SPRIselect Reagent Kit	Beckman Coulter	B23318
TE Buffer, TRIS-EDTA, 1× solution, pH 8.0	Thermo Fisher Scientific	BP24731
Tris 1 M, pH 8.0, RNase-free	Invitrogen	15568025
TrypLE Express Enzyme, phenol red	Gibco	12605010
Tween 20 Surfact-Amps detergent solution (10% solution)	Thermo Fisher Scientific	28320
UltraPure distilled water, DNase/RNase-free	Invitrogen	10977–015
Urea solution, 8 M	MilliporeSigma	51457-100ML

**Critical commercial assays**		

Visium HD, Human Transcriptome, 6.5 mm	10× Genomics	1000675
Dual Index Kit TS Set A, 96 rxns	10× Genomics	1000251

**Deposited data**		

FASTQ files and Space Ranger v.3.1.3 Outputs	GEO (Gene Expression Omnibus)	GSE296623
Associated data	Mendeley Data	https://doi.org/10.17632/sffnnvbdp3
Code	Zenodo	https://doi.org/10.5281/zenodo.15858423

**Experimental models: Cell lines**		

Human cells of your choosing (or mouse if using mouse transcriptome Visium HD kit)		N/A
MDA-MB-231	ATCC	HTB-26
MCF10A	Horizon Discovery	HD 101-005
Primary mammary epithelial cells	ATCC	PCS-600-010
Patient-derived mammary myoepithelial cells	Thi et al.^9^	N/A
Primary mammary fibroblast	ScienCell	7630
Primary HUVEC	AngioProteomie	cAP-0001GFP
Primary macrophage	Celprogen	36070–01

**Other**		

0.2 mL 8-tube strips, PCR tubes	Refer to 10× Genomics Protocol Planner (CG000698) for tested tubes	
1.5 mL, DNA LoBind tubes	Eppendorf	12431021
100 mm, Grip ring, TC treated, sterile, Petri dish	Fisher Scientific	FB012924
1,000 mL Beaker (x 7)		N/A
15 mL Conical tubes	Fisher Scientific	14-959-49B
50 mL Conical tubes	Fisher Scientific	14-432-22
96-well PCR plate	Compatible option for machine	
Aspirating Pipettes		N/A
Bioanalyzer or TapeStation & associated reagents	Refer to 10× Genomics Protocol Planner (CG000698) for tested instruments	
Biosafety cabinet		N/A
Bright-field microscope with camera (i.e. slide scanner)	Refer to 10× Genomics Imaging Guidelines Technical Note (CG000688)	
Cell culture flasks/plates (specific to cells of interest)		N/A
Coverslips, 50 × 24 mm	VWR or equivalent	16004–322
Filtered pipette tips (10, 20, 200, and 1,000 μL)	Fisherbrand or equivalent	N/A
Forceps		N/A
Fume hood		N/A
Ice bucket		N/A
Incubator		N/A
Lab Safe Marker		N/A
Lint-free laboratory wipes	VWR	10835–982
Low Profile Thermocycler Adapter (part of CytAssist Accessory Kit)	10× Genomics	1000499
Magnetic separator (part of CytAssist accessory kit)	10× Genomics	1000499
Microscope slides, Fisherbrand SuperFrost Plus	Fisher Scientific	12-550-15
Mini centrifuge		N/A
Nanodrop or Qubit Fluorometer & associated reagents	Refer to 10× Genomics Protocol Planner (CG000698) for tested instruments	
Pencil or pen		N/A
Razor blade, box cutter, or equivalent		N/A
Reagent reservoir	VWR	41428–958
Real Time qPCR System	Refer to 10× Genomics Protocol Planner (CG000698) for tested instruments	
Serological pipettes (5, 10, and 25 mL)		N/A
Thermal Cycler	Refer to 10× Genomics Protocol Planner (CG000698) for tested instruments	
Thermomixer with 2 mL block	Eppendorf or equivalent	5382000023
Tube racks		N/A

## Materials and equipment

**Table:** Collagen Substrate

Reagent	Working solution	Weight or volume
PureCol	3.2 mg/mL	33.3 μL
UltraPure Distilled Water	–	966.7 μL
**Total**	–	**1000 μL**

Prepare on ice. Pipette mix. Avoid introducing bubbles. Make fresh and use immediately. Total volume provided is the volume needed per microscope slide.

**Table:** Fixation Solution - 4% Paraformaldehyde

Reagent	Working solution	Weight or volume
Paraformaldehyde (PFA)	16%	6.25 mL
Phosphate Buffered Saline	1x	18.75 mL
**Total**	–	**25 mL**

Make fresh and use at room temperature. Total volume provided is the volume needed per microscope slide.

**Table:** Mounting Medium - 85% Glycerol

Reagent	Working solution	Weight or volume
Glycerol	100%	127.5 μL
Nuclease-free water	–	22.5 μL
**Total**	–	**150 μL**

Invert to mix. Avoid introducing bubbles. Briefly centrifuge until no bubbles remain. Total volume provided is the volume needed per microscope slide.

## Step-by-step method details

### Substrate preparation

**Timing: approximately 2.5 h**

This section details preparing the microscope glass slide to receive adherent cells.

**CRITICAL:** Proper sterile technique must be followed at all times to ensure successful outcomes. Clean surfaces with 70% ethanol. Keep everything capped whenever not actively accessing it. Do not reach over an open dish or container. Use sterile consumables.

1. In the biosafety cabinet, use sterile forceps to transfer one of the sterilized microscope slides to the 100 mm petri dish.

***Note:*** If the optional reference guide steps above were performed, use that Petri dish for the remainder of the protocol.

**Figure 2:**
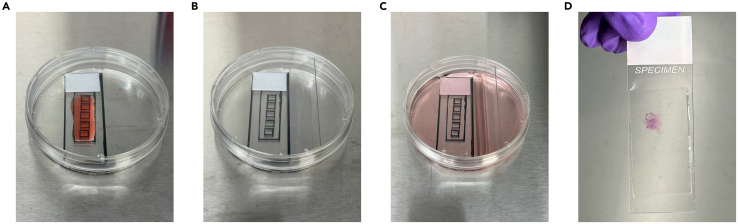
Culture preparation and sample generation (A) Spreading of collagen throughout viable region. Red food coloring used for illustrative purposes. (B) Addition of second microscope slide to prevent the sample from moving around. (C) Cell culture medium addition, ensure sample is completely submerged. (D) Hematoxylin and eosin stained sample with coverslip in place, void of bubbles.

2. Pipette ∼750 μL of the prepared PureCol collagen substrate solution onto the center of the slide, covering the entire viable region (Figure 2A). Carefully spread with pipette tip if needed.

***Note:*** Other common culture coating solutions such as gelatin, Matrigel, fibronectin, laminin, Poly-L-Lysine, or Poly-D-Lysine may also be compatible but have not been tested.

3. Return the lid to the petri dish and incubate at room temp for 2 h. Do not disturb.

**CRITICAL:** Do not exceed 3 h. Extended incubation times will lead to collagen drying out and flaking off the slide.

4. Carefully add 1× PBS to the petri dish, along the side of the wall, until the glass slide is completely submerged (∼25 mL).
5. Carefully aspirate without disrupting the coated slide.
6. Repeat steps 4 and 5 to wash for a total of 3 times.
7. Immediately add ∼25 mL 1× PBS to the petri dish, ensuring the slide is completely covered, to prevent the collagen from dehydrating.
8. Using sterile forceps, transfer the second sterile microscope slide to the petri dish being careful to not touch the surface of the collagen coated slide. Place at an angle to hold the coated slide in place (Figure 2B).
9. Carefully aspirate PBS and replace with complete cell culture medium (Figure 2C).

***Note:*** We recommend including 2 μL Primocin per mL of culture medium to prevent contamination. Concentration may vary depending on cell type. Consult product manual for recommendations.

10. Cover petri dish and store in a 37°C incubator while harvesting the cells.

### Cell harvest, deposition, and culture

**Timing: varies; 4 h to week(s), depending on desired culture duration**

This section details sample generation and growth. During the development of this protocol, the cells were patterned via single-cell bioprinting as previously reported,^8^ and cultured for 24 h. Alternatively, cells can be seeded onto the microscope slide surface by pipette and grown for the desired duration.

**CRITICAL:** Proper sterile technique must be followed at all times to ensure successful outcomes. Clean surfaces with 70% ethanol. Keep culture vessels and reagents covered/closed whenever not actively accessing them. Never reach over an open container. Use sterile consumables.

11. Harvest cells.
  a. Aspirate media from culture flask/dish.
  b. Add enough TrypLE to cover the cells.
    ***Note:*** Other cell detachment solutions may be used in place of TrypLE, such as Trypsin-EDTA, Accutase, or Dispase. Consult product manuals for best practices for the cells of interest.
  c. Observe the cells under the microscope to look for detachment. Gently tap the side of the flask/dish to promote detachment.
    ***Note:*** Cells should detach within 2–3 min with TrypLE. If they are not detaching, place culture vessel in the incubator for 1 min, tap the side of the flask, then check again under the microscope. Depending on cell type, a cell scraper may also be used.
    **CRITICAL:** Do not overexpose cells to enzymatic detachment solutions as this will impact viability.
  d. Add equal volume of complete medium to the flask to stop the enzymatic reaction.
  e. Transfer contents of the culture vessel to an appropriate size conical tube.
  f. Centrifuge at 300 × g for 3 min at room temperature, or recommended setting for the cells of interest.
  g. Aspirate supernatant.
  h. Resuspend cells in complete medium.
  i. Count cells and adjust volume to achieve the desired concentration, centrifuging and resuspending again if necessary.
    i For single-cell printing: 2 × 10^6^ cells/mL.
    ii For bulk cell dispensing via the single-cell printer: 8 × 10^6^ cells/mL
    iii For pipette seeding: concentration depends on experimental conditions and desired outcome
12. Deposit cells onto the collagen-coated microscope slide.
  a. Details for single-cell printing can be found in Helms et al.^8^
  b. To pipette seed, dispense desired concentration of cells above the substrate. Gently swirl the dish to promote even distribution.
13. Carefully return sample to the incubator. Culture for desired duration and perform any desired experimentation (i.e. drug treatment, wound assay, etc.).

***Note:*** Cells should be cultured for a minimum of 2 h to ensure adequate adhesion. The Visium HD protocol requires many liquid exchange steps that risk washing the cells away. In this example, we culture the cells for 24 h. Depending on cell behavior, extended culture periods may also be problematic if cells aggregate into 3D structures. We have not determined what the upper thickness threshold is for successful probe transfer in the CytAssist. However, the Visium HD manual recommends cutting tissues into sections 3–20 μm thick.

***Note:*** If this is the first time performing the Visium HD spatial gene expression library construction, we recommend reviewing the Visium HD Spatial Gene Expression User Guide (CG000685), especially the Tips & Best Practices and Visium CytAssist sections, while the sample is growing. Practice assembling and disassembling the Tissue Slide Cassette with a blank slide as outlined in the Visium CytAssist Training Kit User Guide (CG000549). Once the sample is fixed, there will be no safe stopping points until partway into the Visium HD protocol. It is critical all steps and time limits are followed to successfully construct a library with the highest quality data.

### Sample fixation and permeabilization

**Timing: 1.5 h**

This section details the process for fixing and permeabilizing the sample on the microscope slide. To preserve RNA quality, we recommend proceeding immediately through to the Visium HD Spatial Gene Expression User Guide (CG000685). Probe hybridization runs overnight (16–24 h) and is the first hands-off point. The first safe stop and store point is after the completion of probe ligation, at which point the sample can be stored at 4°C for up to 24 h. Please plan accordingly.

**CRITICAL:** During all subsequent steps it is critical to not disturb the sample. Pipetting directly onto the sample can cause detachment.

14. In a fume hood, transfer 45 mL 100% methanol to a 50 mL conical tube.
15. Submerge the conical tube in an ice bucket up to the lid.
  a. Chill for a minimum of 30 min. This will be used in steps 25–26.
16. In a biosafety cabinet, carefully aspirate media from culture vessel.
17. Without disturbing the sample, add ∼25 mL of 1× PBS, enough to completely cover the sample. Aspirate along the edge of the dish without disturbing the sample.
18. Repeat two more times for a total of 3 washes.
19. Aspirate PBS and add ∼25 mL 4% paraformaldehyde (PFA), enough to completely cover the sample.
20. Incubate at room temperature for 30 min.
21. Now that the sample is fixed, transfer to the fume hood and remove PFA, ensuring proper waste disposal.
22. Without disturbing the sample, add ∼25 mL of 1× PBS to wash.
23. Remove and repeat two more times for a total of 3 washes. Again, ensure proper waste disposal.
24. Remove slide from PBS and gently tap the edge of the slide onto a lint-free laboratory wipe. Wipe excess PBS from the back of the slide. Do not touch the section and do not allow the sample to dry out.
25. Immerse the slide into the conical tube containing chilled 100% methanol prepared in steps 14–15. Keep tube buried in ice.
26. Incubate on ice for 1 h.
27. Remove slide from methanol and return to petri dish.
28. Without disturbing the sample, add ∼25 mL of 1× PBS to wash.
29. Remove and repeat two more times for a total of 3 washes. Again, ensure proper waste disposal.
30. Keep sample hydrated at all times, submerged in 1× PBS.

**CRITICAL:** Proceed immediately through the following major steps until probe hybridization of the Visium HD Spatial Gene Expression User Guide (CG000685, page 50, step n, of Rev C). To preserve RNA quality, we do not recommend storing a fixed sample.

### Staining and imaging

**Timing: 30 min to 2 h**

This section details the steps to perform hematoxylin and eosin (H&E) staining. Immediately following staining, a high resolution, stitched image will be taken of the entire slide. Currently, Visium HD is offered for a 6.5 x 6.5 mm capture area. Only one 6.5 x 6.5 mm region of a microscope slide can be transferred. It is important that the imaging aligns with what will be sequenced, as this image will be imported into the sequencing alignment pipeline (Space Ranger) and used during data analysis. We therefore recommend oversampling during image (i.e., image the majority of the slide) and create the cropped region of interest image that aligns with the CytAssist image before running Space Ranger. Please consult 10x Genomics, Visium HD Spatial Applications Imaging Guidelines Technical Note (CG000688) for additional imaging guidance.

***Note:*** Immunofluorescence (IF) can also be performed on the sample prior to H&E staining. Please be aware, depending on the integrity of your 2D culture/tissue and the adhesion to the slide, the extra liquid exchanges of the IF protocol may increase likelihood of sample detachment. Refer to 10× Genomics, Visium HD Fixed Frozen Tissue Preparation Handbook (CG000764), Section 3, for detailed instructions, including approved reagents, to perform IF.

**CRITICAL:** Be extra gentle during liquid exchanges to prevent sample detachment. Never touch the sample; it is better to have a little excess liquid remaining on the sample than to disturb/damage the sample trying to remove all liquid.

31. Set up seven 1,000 mL beakers, each filled with 800 mL Milli-Q or UltraPure water. Label 1-7.
32. Remove slide from PBS and gently tap the edge of the slide onto a lint-free laboratory wipe to remove excess PBS. Do not touch sample!
33. Place slide on a flat, clean, nonabsorbent work surface.
34. Gently add 1 mL of hematoxylin onto the slide to uniformly cover the sample.
35. Incubate for 1 min at room temperature.
36. Remove hematoxylin by tilting the slide over a discard jar (i.e. 50 mL conical tube) and gently tap the edge of the slide onto a lint-free laboratory wipe to remove excess.
37. Immerse the slide into Water Beaker #1 and gently dunk 5 times.
38. Immerse the slide into Water Beaker #2 and gently dunk 15 times.
39. Immerse the slide into Water Beaker #3 and gently dunk 15 times.
40. Gently tap the edge of the slide onto a lint-free laboratory wipe and wipe excess water from the back of the slide. Do not touch the section! Some remaining water is okay.
41. Return the slide to a flat, clean, nonabsorbent work surface.
42. Add 1 mL bluing buffer onto the slide to uniformly cover the sample.
43. Incubate for 1 min at room temperature.
44. Remove bluing buffer by tilting the slide over a discard jar (i.e. 50 mL conical tube) and gently tap the edge of the slide to a lint-free laboratory wipe to remove excess.
45. Immerse the slide into Water Beaker #4 and gently dunk 15 times.
46. Gently tap the edge of the slide onto a lint-free laboratory wipe and wipe excess water from the back of the slide. Do not touch the section! Some remaining water is okay.
47. Add 1 mL of fresh eosin (do not use diluted eosin) onto the slide to uniformly cover the sample.
48. Incubate for 1 min at room temperature.
49. Remove eosin by tilting the slide over a discard jar (i.e. 50 mL conical tube) and gently tap the edge of the slide onto a lint-free laboratory wipe to remove excess.
50. Immerse the slide into Water Beaker #5 for 30 seconds.
51. Immerse the slide into Water Beaker #6 and gently dunk 10 times.
52. Gently tap the edge of the slide onto a lint-free laboratory wipe and wipe excess water from the back of the slide. Some remaining water is okay.

**CRITICAL:** Do not air dry the slide.

53. Return the slide to a flat, clean, nonabsorbent work surface.
54. Using a P1000 pipette tip, add 100–150 μL mounting medium (85% glycerol) to cover the entire sample evenly. DO NOT INTRODUCE BUBBLES.

**CRITICAL:** Do not use any other type of mounting medium or sealant (i.e. Cytoseal or nail polish) as this can impair downstream library construction.

55. Slowly apply the coverslip by angling from one end of the slide and gently lying flat without introducing bubbles (Figure 2D).

**CRITICAL:** Do not move the coverslip or disturb the tissue!

**Figure 3:**
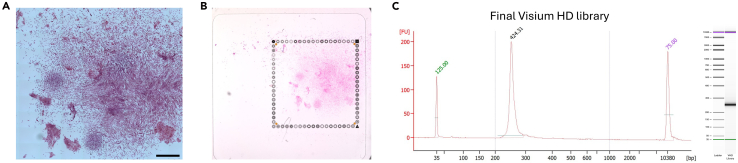
Visium HD library preparation and quality control (A) Hematoxylin and eosin stain of the resulting 2D single-cell print on a collagen coated microscope slide. Scale bar = 1 mm. (B) CytAssist image during probe transfer from the cells to the Visium HD capture area. (C) Electropherogram (Agilent BioAnalyzer 2100, High Sensitivity DNA Assay) of the final Visium HD library.

56. If necessary, use the edge of a lint-free laboratory wipe to wick away excess mounting medium along the edges of the slide. Do not move the coverslip or disturb the tissue!
57. Immediately proceed with imaging. Transport the slides laid flat – do not tilt.
  a. Image the sample at 20–40× magnification using bright-field imaging settings (Figure 3A). Refer to the 10x Genomics, Visium HD Spatial Applications Imaging Guidelines Technical Note (CG000688) for additional imaging guidance.
58. Immediately following imaging, immerse the slide with the long edge of the slide parallel to the bottom of the beaker, into Water Beaker #7.
59. Hold the slide in the water until the coverslip slowly separates and falls off the slide.

**CRITICAL:** Do not shake the slide or touch the coverslip. The coverslip should gently detach, or you risk sample damage and/or detachment.

60. Once the coverslip has detached, gently dunk the slide in the same Water Beaker #7 30 times to ensure all mounting medium has been removed.
61. Gently tap the edge of the slide onto a lint-free laboratory wipe and wipe excess water from the back of the slide. Do not touch the sample! Some remaining water is okay.
62. Return the slide to a flat, clean, nonabsorbent work surface.
63. Air dry for 5 min.
64. Incubate the slide on the low profile thermocycler adapter, with the lid of the thermal cycler open, for 3 min at 37°C.
65. Proceed immediately to destaining.

### Destaining and decrosslinking

**Timing: 1.5 h**

This section covers destaining and decrosslinking, following the Visium HD FFPE Tissue Preparation Handbook (CG000684, Rev A). At the time of this protocol development, the fixed frozen (FxF) tissue manual had not been released. Current (Rev C) FxF and FFPE handbooks follow the same destaining and decrosslinking protocols.

66. Perform all destaining (Section 2.6, pages 55–58) and decrosslinking (Section 2.7, pages 59–60) steps as instructed in the Visium HD Fixed Frozen Tissue Preparation Handbook (CG000764, Rev C).

### Construction of Visium HD library

**Timing: 3+ days**

Carefully follow each step of the Visium HD Spatial Gene Expression User Guide (CG000685) to construct a spatial gene expression library. Please note, revision A of CG000685 was used at time of protocol development. Refer to the manual introduction for a timeline overview of the process including safe stopping points (page 15 of Rev C).

67. Perform all steps according to the Visium HD Spatial Gene Expression User Guide (CG000685). The protocol begins at the section titled “Step 1: Probe Hybridization” (page 46 of Rev C).

***Note:*** Consult the Visium HD Spatial Gene Expression User Guide (CG000685) for troubleshooting, tips, and best practices related to library construction.

## Expected outcomes

Successful completion of the protocol yields a high resolution hematoxylin and eosin (H&E) image of the generated sample (Figure 3A), a bright-field image captured by the CytAssist instrument during probe transfer (Figure 3B), and a Visium HD spatial gene expression library (Figure 3C) ready for sequencing. Sequencing guidelines and recommendations can be found in the Visium HD Spatial Gene Expression User Guide (CG000685, pages 91–102 of Rev C).

**Figure 4:**
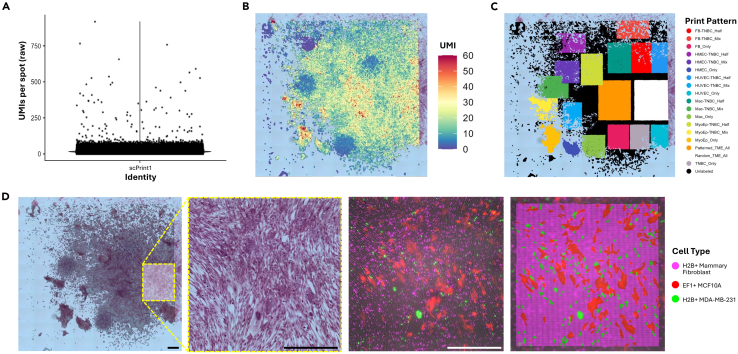
Basic spatial transcriptomic data visualization (A) Number of unique molecular identifiers (UMIs) per capture spot. Raw data, 8 μm binning. (B) Spatial distribution of UMIs overlayed on the H&E image. Raw data, 8 μm binning, cropped to display values between 0 and 60. (C) Sample annotation across the capture area. (D) Cell type annotation facilitated by corresponding fluorescence image of cells that were transduced with unique reporters. H2B-iRFP+ human primary mammary fibroblasts, EF1-tdTomato+ MCF10A mammary epithelial cells, and H2B-mVenus+ MDA-MB-231 breast cancer cell line. Scale bar = 500 μm.

It is important to note that it is unlikely an in vitro sample will have the same exact gene expression profile as a native tissue. As such, we expect reduced expression levels (measured as unique molecular identifier [UMI] counts) of genes associated with tissue-specific functions (Figure 4A). In addition, low cell seeding density allows cells to spread across many 2 μm x 2 μm barcoded spots (Figure 4B), further reducing the average UMIs per spot.

A major advantage of performing spatial transcriptomics on plated cells or engineered tissues, compared to retrospective analysis of resected tissue sections, is that the starting cell populations are defined. In this demonstration, single-cell bioprinting^8^ was used to precisely control the initial spatial location of each cell (Figure 4C). When combined with unique fluorescent reporters, the engineered design provides ground truth for benchmarking both cell identity and spatial position (Figure 4D). This experimental precision enables high-resolution analysis of how spatial arrangement and local cell-cell interactions shape gene expression, allowing systematic dissection of the spatial mechanisms underlying development, disease progression, and therapeutic response.

Similar to single-cell RNA-sequencing of in vitro models, spatial transcriptomic profiling of in vitro models will open new opportunities to systematically investigate cause and effect relationships in cell-cell interactions driving health and disease.

## Quantification and statistical analysis

Raw sequencing data (FASTQ files) are processed using the 10x Genomics Space Ranger pipeline, which outputs gene expression matrices, spatial metadata, aligned tissue images, and quality control metrics. Space Ranger can be run either on 10x Genomics’ free cloud platform,^10^ on a server, or locally if your workstation meets the hardware demands.^11^ 10x Genomics has provided step by step instructions for downloading the software, reference transcriptome, and probe set.^12^

Space Ranger requires the following file inputs: FASTQ files, reference transcriptome, probe set, H&E or immunofluorescence image, CytAssist image, slide number, and capture area. While the pipeline will automatically align the H&E image and the CytAssist image, we recommend performing manual alignment^13^ in Loupe Browser^14^ (10x Genomics). Proper alignment is critical for downstream analysis. Since cell cultures and engineered tissues are considered unusual data inputs, automated alignment may underperform. We recommend placing all input files into a single folder with clear names and no spaces in the file name. Once the pipeline successfully finishes, inspect the web summary and metrics .csv. Proceed with downstream analysis using tools such as Loupe Browser,^15^ Seurat,^16^^,^^17^ Squidpy,^18^^,^^19^ or equivalent.^20^

**Figure 5:**
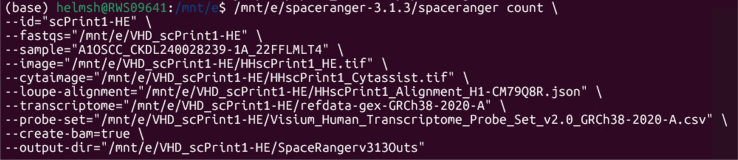
Example command-line execution of the Space Ranger (v.3.1.3) count pipeline

In this demonstration, the library was sequenced on an Illumina NovaSeq X Plus with a depth of approximately 200 million reads per sample. We ran Space Ranger v3.1.3 locally via Windows Subsystem for Linux (WSL) with the data saved at the following path: /mnt/e/VHD_scPrint1 (Figure 5). The slide and area commands were not included since this information is included in the Loupe alignment file.

**Figure 6:**
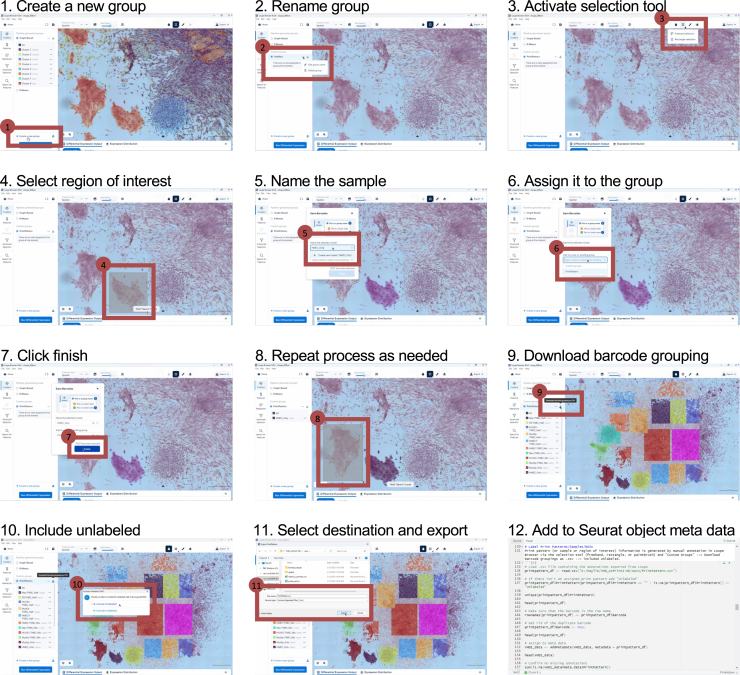
Barcode annotation in Loupe Browser and addition to Seurat object metadata

The resulting 8 μm binned .cloupe file was opened in Loupe Browser to annotate the samples displayed in Figure 4C. Custom groups were created with the selection tool and all barcodes, included unlabeled, were exported (Figure 6). The Space Ranger outputs were loaded into Seurat (v5) and the Loupe custom groupings were added to the meta data of the Seurat object. The complete code has been made available.

## Limitations

Much remains unknown of the specifics regarding probe release, probe transfer, probe capture, and the internal CytAssist instrument design specifications as these details are all protected intellectual property of 10x Genomics. We do know that the “probe release mix” containing “perm enzyme B″ spreads between the sample slide and the Visium HD capture slide through capillary action and that the probes are “transferred via mass transport”. Sample thickness, substrate properties, and variations in sample topography are all likely to interfere with the spreading of the probe release enzyme, diffusion of enzyme, transport of probe, and resulting probe capture. The Visium HD Spatial Gene Expression User Guide (CG000685, Rev C) states: “Inappropriate flow of reagents during a Visium CytAssist experiment run may result in transcript mislocation.” The distance between the Visium HD capture slide and the microscope slide containing the sample when the CytAssist is closed is unknown. However, we do know that the recommended tissue section thickness is 3–10 μm for FFPE (CG000684, Rev C) and 10–20 μm for fixed frozen (CG000764, Rev C). Therefore, we advise keeping the sample thickness below 20 μm. In this protocol we use PureCol, a bovine collagen substrate. Other conventional culture coatings, such as gelatin, Matrigel, fibronectin, laminin, Poly-L-Lysine, or Poly-D-Lysine, may perform better or comparatively but have not been tested.

## Troubleshooting

### Problem 1

Low assay performance (related to Step 67).

### Potential solution

Closely follow all steps of the Visium HD Spatial Gene Expression User Guide (CG000685) as listed, paying special attention to the numbers of washes, timing of critical steps, and complete reagent removal. For example, washes following probe hybridization and probe ligation have significant impact on the fraction of targetable reads. Performing less washes than instructed, reducing the time of the wash, or inadequate removal of solution will lead to low performance assay. Refer to the Visium HD Spatial Gene Expression User Guide (CG000685, pages 26–27 of Rev C) for visual demonstration of best practices for reagent swapping. We recommend practicing with a colored solution (i.e. water with food coloring) prior to starting the protocol.

There are also several critical time sensitive steps during probe transfer. Over-drying of the capture slide, under-drying of the capture slide, delayed start of the CytAssist run following perm enzyme B addition to the probe release mix, and delayed removal of the capture slide after probe transfer will all decrease assay performance. We recommend carefully studying the protocol ahead of time. It may also be helpful to have a second person assisting.

Overconfluent or multilayer cultures can also impair probe penetration, fiducial registration in the CytAssist, or probe capture. Reduce initial cell density or culture period to avoid cell aggregation into multilayered structures (Step 12).

### Problem 2

UMI heatmap has areas under the tissue with 0 UMIs (related to Step 67).

### Potential solution

The most likely causes are bubble entrapment, uneven flow of the probe release mix, or debris on the capture slide (i.e. lint). Insufficient drying of the capture slide or tissue before closing the CytAssist may result in uneven flow or bubble entrapment. Carefully inspect capture slide, increasing the drying time as necessary. To speed up drying of the tissue, gently waft/flick slide back and forth (demonstration shown on page 72 of the Visium HD Spatial Gene Expression User Guide, CG000685 Rev C). A lint-free laboratory wipe can be used along the edges of the slide, but it is critical to not touch the tissue. Also ensure the proper volume of probe release enzyme is dispensed into the spacer well and no bubbles are present. An uneven or multilayer sample can also cause inappropriate flow of the probe release mix across the sample. Adjust cell seeding density to reduce aggregation (Step 12). Lastly, to reduce the chance of debris landing on the capture slide, avoid reaching directly over the slide while aligning the tissue.

### Problem 3

UMIs are detected in spots where there are no cells (related to quantification and analysis).

### Potential solution

Inappropriate flow of reagents during the probe transfer, caused by insufficient capture area/tissue drying or a multilayer sample, can cause transcript mislocalization. Misalignment of reads to the image may also be an artifact of the Space Ranger pipeline. Follow the instructions within Loupe Browser (10x Genomics software) to manually align the full resolution H&E image with the CytAssist image prior to running Space Ranger. Additionally, it is possible that RNA-containing materials such as exosomes, cell debris, or secreted factors were deposited into the collagen substrate during culture. These residual components may bind probes during hybridization and be transferred to the capture area, leading to UMIs being detected in regions with no cells.

### Problem 4

Sample detaches from the glass microscope slide (related to Steps 13–67).

### Potential solution

Sample detachment can occur due to high cell confluency, suboptimal substrate adhesion, or physical disruption during reagent handling. Reduce initial cell seeding density (Step 12) or shorten the culture duration as overly confluent monolayers are more likely to detach in sheets. If adhesion remains suboptimal, switching to an alternative substrate, such as gelatin, Matrigel, fibronectin, laminin, Poly-L-Lysine, or Poly-D-Lysine, may improve cell attachment (Step 2). While this protocol was validated using a PureCol substrate, the Visium HD Spatial Gene Expression User Guide (CG000685) recommends Schott Nexterion Slide H – 3D Hydrogel Coated Slides to minimize tissue detachment, suggesting that other substrates are likely compatible with the downstream library preparation. Finally, carefully add and remove solutions without disrupting the tissue and never allow the sample to dry out as this will increase the likelihood of detachment.

### Problem 5

Tissue damaged during library preparation.

### Potential solution

As it is critical to remove all solutions between steps to preserve assay performance, there is a high likelihood of accidentally touching the tissue with the pipette tip. We recommend leaving the bottom right corner of the tissue slide cassette clear of tissue, if possible, by adjusting the gasket alignment during cassette loading (Step 66).

## Resource availability

### Lead contact

Further information and requests for resources and reagents should be directed to and will be fulfilled by the lead contact, Luiz E. Bertassoni (bertasso@ohsu.edu).

### Technical contact

Technical questions on executing this protocol should be directed to and will be answered by the technical contact, Haylie R. Helms (helmsh@ohsu.edu).

### Materials availability

No new materials were generated in this study.

### Data and code availability

The dataset generated during this study has been deposited in the National Center for Biotechnology Information (NCBI) Gene Expression Omnibus and is accessible through GEO Series accession number GSE296623: https://www.ncbi.nlm.nih.gov/geo/query/acc.cgi?acc=GSE296623. All data for Figures 3 and 4 have been deposited to Mendeley Data: https://doi.org/10.17632/sffnnvbdp3. The code used to generate the basic data visualization in Figure 4 is available at https://zenodo.org/records/15858423.

## Acknowledgments

We thank the following 10× Genomics team members for their helpful discussions during the conceptualization of this protocol: Dr. Lionel Brooks, Spatial Field Application Scientist – Tissue Specialist; Dr. Eliot T. Smith, Spatial Science and Technology Advisor; and Dr. Wendy Love, Account Executive. This work was supported by funding from the Cancer Early Detection Advanced Research Center, the Knight Cancer Institute, and the Oregon Health and Science University (Full 2023-1745), awarded to H.R.H. and L.E.B. Institutional support was provided by the OHSU Knight Cancer Institute NCI Cancer Center Support Grant (P30CA069533) for the Advanced Light Microscopy Core (RRID: SCR_009961). L.E.B. acknowledges support from the National Institute of Dental and Craniofacial Research (R01DE035326), the OHSU Silver Family Innovation Award, the M.J. Murdock Charitable Trust, the Knight Cancer Institute Pilot Award, and the Cancer Early Detection Advanced Research Center (Full 2023-1719). H.R.H. also acknowledges support from the International Alliance for Cancer Early Detection (ACED) PhD Fellowship and the Achievement Rewards for College Scientists (ARCS) Foundation Oregon. The International Alliance for Cancer Early Detection is a partnership between Cancer Research UK, the Dana-Farber Cancer Institute, The University of Manchester, the German Cancer Research Center (DKFZ), University College London, the Knight Cancer Institute at OHSU, and the University of Cambridge.

## Author contributions

H.R.H. conceptualized the method, performed the experiments, and wrote the original manuscript. L.E.B. supervised and reviewed the manuscript.

## Declaration of interests

The authors declare no competing interests.
